# Differences in the Time Course of Learning for Hard Compared to Easy Training

**DOI:** 10.3389/fpsyg.2013.00110

**Published:** 2013-03-07

**Authors:** Adrian Garcia, Shu-Guang Kuai, Zoe Kourtzi

**Affiliations:** ^1^School of Psychology, University of BirminghamEdgbaston, Birmingham, UK; ^2^Laboratory for Neuro- and Psychophysiology, Katholieke Universiteit LeuvenLeuven, Belgium

**Keywords:** perceptual learning, form perception, psychophysics, Glass patterns

## Abstract

Learning is known to facilitate performance in a range of perceptual tasks. Behavioral improvement after training is typically shown after practice with highly similar stimuli that are difficult to discriminate (i.e., hard training), or after exposure to dissimilar stimuli that are highly discriminable (i.e., easy training). However, little is known about the processes that mediate learning after training with difficult compared to easy stimuli. Here we investigate the time course of learning when observers were asked to discriminate similar global form patterns after hard vs. easy training. Hard training required observers to discriminate highly similar global forms, while easy training to judge clearly discriminable patterns. Our results demonstrate differences in learning and transfer performance for hard compared to easy training. Hard training resulted in stronger behavioral improvement than easy training. Further, for hard training, performance improved during single sessions, while for easy training performance improved across but not within sessions. These findings suggest that training with difficult stimuli may result in online learning of specific stimulus features that are similar between the training and test stimuli, while training with easy stimuli involves transfer of learning from highly to less discriminable stimuli that may require longer periods of consolidation.

## Introduction

Practice is known to improve a wide range of visual perceptual skills from low-level feature discrimination (e.g., orientation Fiorentini and Berardi, 1980; Matthews et al., 1999; motion direction Ball and Sekuler, 1987; Liu, 1999; Lu et al., 2006; texture Karni and Sagi, 1991, 1993; Ahissar and Hochstein, 1996) to high-level shape processing and object recognition (Furmanski and Engel, 2000; Golcu and Gilbert, 2009), demonstrating the remarkable plasticity of the adult visual system (for reviews, see Ahissar, 2001; Fine and Jacobs, 2002; Fahle, 2004; Kourtzi, 2010; Sagi, 2011). Designing effective training programs is critical for applications in rehabilitation. Training task difficulty has been identified as one of the main factors that contribute to training outcome (Ahissar and Hochstein, 1997; Liu and Weinshall, 2000; Liu et al., 2012). It is widely believed that supervised training (i.e., training with feedback) on difficult tasks that require discrimination of highly similar stimuli improves participants’ performance (Ball and Sekuler, 1987; Shiu and Pashler, 1992; Fahle and Edelman, 1993; Herzog and Fahle, 1997; Dwyer et al., 2004; Seitz et al., 2006; Aberg and Herzog, 2012). However, there is accumulating evidence that training on easy discrimination tasks (i.e., when stimuli are clearly discriminable) may also facilitate performance in perceptual judgments (Ahissar and Hochstein, 1997; Rubin et al., 1997; Liu and Weinshall, 2000; Jeter et al., 2009; Liu et al., 2010, 2012). Although these studies have suggested that hard vs. easy training may relate to different learning processes, previous work has focused on assessing the final outcome of training rather than the time course of learning. Investigating the time course of learning-dependent improvements is important for understanding the processes that underlie learning based on hard vs. easy training.

**Figure 1:**
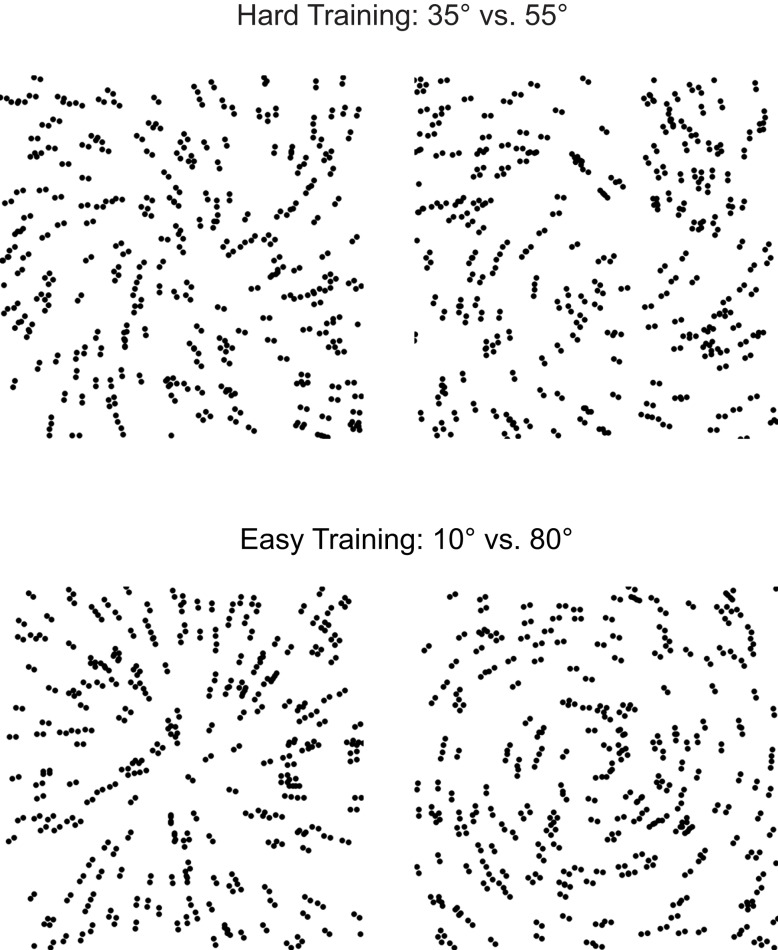
**Glass pattern stimuli**. Schematics of radial and concentric Glass patterns used for hard (35° vs. 55°) vs. easy (10° vs. 80°) training.

To address this question, we designed a stimulus space and a paradigm that allowed us to compare the time course of behavioral improvement during training on a hard vs. easy shape discrimination task. We used parametric manipulations of Glass patterns that comprise oriented dot dipoles (Figure 1). For these stimuli, small local changes to dot patterns have a predictable influence on the perception of global forms (concentric vs. radial patterns). We manipulated the difficulty of the training task by varying the similarity between global forms, using linear morphing between concentric and radial patterns. Hard training involved training on similar patterns, while easy training involved training on highly discriminable patterns. We assessed training outcome by testing observers on the discrimination of similar patterns without feedback. To monitor improvement of behavioral performance during hard vs. easy training we interleaved training and test blocks within each session.

Our results demonstrate differences in the time course of learning for hard vs. easy training. In particular, training on a hard discrimination resulted in stronger behavioral improvement than training on an easy discrimination. Interestingly, for hard training performance improved within the time course of a single session, while for easy training performance improved across but not within sessions. These findings suggest differences in the processes that underlie learning based on hard vs. easy training. Training on a difficult task supports continuous and strong improvement in the discrimination of specific features that are similar between training and test (i.e., observers are asked to discriminate highly similar stimuli in both the training and test). However, training on an easy task requires transfer of learning, as stimulus features differ between the stimuli used for training (i.e., highly discriminable) and test (i.e., highly similar). As a result, behavioral improvement is lower following easy than hard training and may require consolidation across sessions.

## Materials and Methods

### Participants

Thirty-six observers (16 male, 20 female, mean age 24 ± 6) participated in the four experiments. None of the participants had prior experience with the stimuli or the study protocol. All participants had normal or corrected-to-normal vision, gave written informed consent and were paid for their participation. The study was approved by the University of Birmingham ethics committee.

### Stimuli

Glass pattern stimuli (Glass, 1969) were used, as previously described (Li et al., 2009). In particular, stimuli comprised of white dot pairs (i.e., dipoles) displayed within a square aperture (7.7° × 7.7°) on a black background (100% contrast). Each dipole comprised two (2.3 × 2.3 arc min^2^) dots with 16.2 arc minutes separation between them. These parameters were chosen based on pilot psychophysical studies and in accordance with previous work (Wilson and Wilkinson, 1998) showing that coherent form patterns are reliably perceived for these parameters.

We generated concentric and radial Glass patterns by placing dipoles tangentially (concentric stimuli) or orthogonally (radial stimuli) to the circumference of a circle centered on the fixation dot (Figure 1). Further, we generated intermediate patterns between these two Glass pattern types by parametrically varying the spiral angle of the pattern from 0° (radial pattern) to 90° (concentric pattern). For each dot dipole, the spiral angle was defined as the angle between the dot dipole orientation and the radius from the center of the dipole to the center of the stimulus aperture. To ensure that participants learnt to discriminate global shapes rather that local features, we jittered randomly the spiral angle (±2.5°) of each presented stimulus. In addition, we generated a new pattern for each stimulus presented in a trial, resulting in stimuli that were locally jittered in their position. All stimuli were generated using Psychtoolbox 3 software in conjunction with Matlab and were presented on a 21″ CRT monitor (1280 × 1024, 85 Hz frame rate). Experiments were conducted in a dark room and the viewing distance was kept constant at 47 cm.

### Procedure

Four experiments were conducted. In Experiment 1, 16 participants were randomly assigned to an easy or a hard training group. In the hard training group, observers were trained to discriminate Glass patterns with spiral angles of 35° (radial) and 55° (concentric). In the easy training group, observers were trained to discriminate Glass patterns with spiral angles of 10° and 80°. Participants in both the hard and easy training groups were tested with spiral angles of 35° and 55°. Observers participated in three sessions conducted on consecutive days. Each session comprised four test blocks without feedback and three training blocks with auditory error feedback. The test and training blocks were interleaved during the session; the session started and ended with a test block. This design allowed us to characterize the time course of learning during each session rather than measuring performance only before and after training. Each block consisted of 200 trials. In each trial, a stimulus image was presented for 200 ms and participants were asked to judge whether the stimulus was radial (left mouse click) or concentric (right mouse click). To avoid participant fatigue, participants took breaks of a minimum of 60 s after each 100 trials with a longer break of 180 s half way through the session.

Experiment 2 tested whether lower performance after easy compared to hard training was due to the limited number of training sessions. Eight participants were trained for six to eight consecutive sessions. The same protocol and stimulus parameters were followed as in Experiment 1. For each individual participant, training stopped after performance had saturated.

Experiment 3 (*n* = 5) controlled for the possibility that performance differences between the hard and easy training groups in Experiment 1 were due to the fact that participants in the hard training group were trained and tested with stimuli presented at the same spiral angle (35° vs. 55°). Participants were trained with stimuli presented at spiral angles of 40° vs. 50° and tested with stimuli presented at spiral angles 35° vs. 55°. That is, the training stimuli were more difficult to discriminate than the test stimuli.

Experiment 4 (*n* = 7), controlled for the possibility that improved performance could result from learning during the test blocks rather than from supervised training. Participants were tested on stimuli presented at spiral angle of 35° vs. 55° (four test blocks as in Experiment 1) but were not trained with feedback on any additional blocks.

## Results

### Experiments 1 and 2: Behavioral improvement following hard vs. easy training

**Figure 2:**
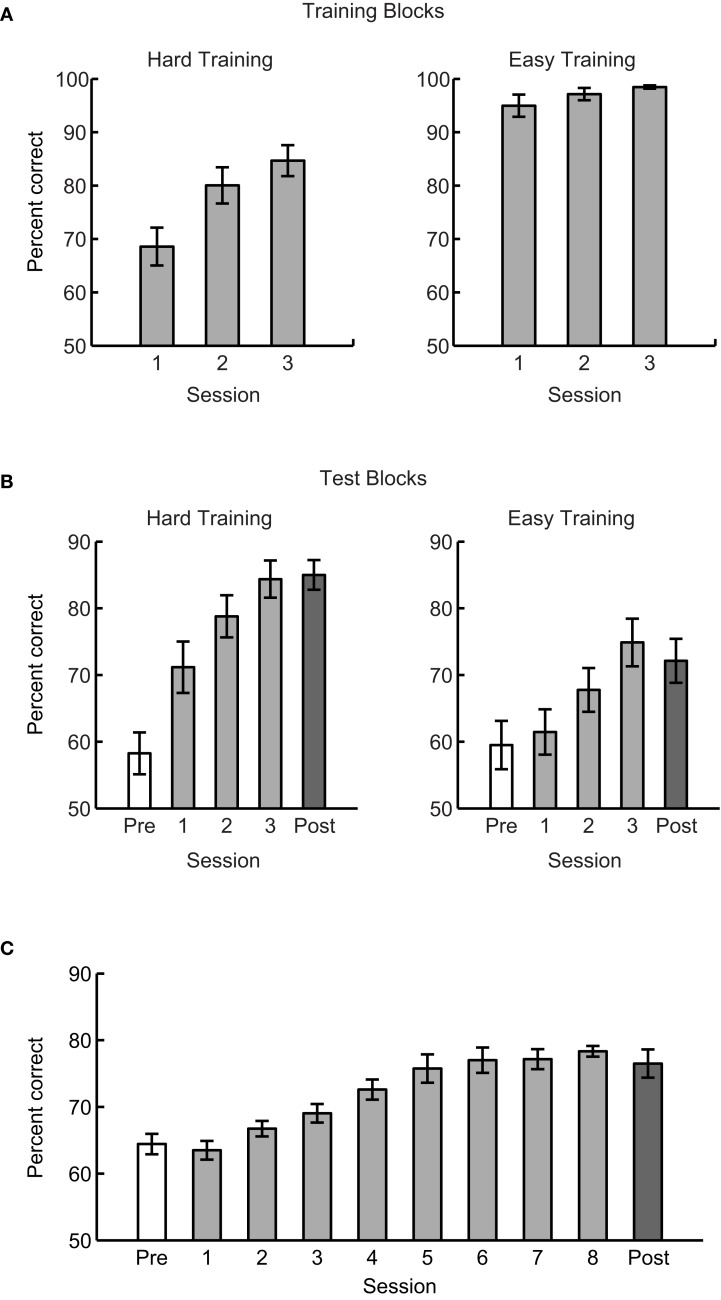
**Experiments 1 and 2: mean behavioral performance across participants for (A) training blocks in Experiment 1, (B) test blocks in Experiment 1, (C) test blocks in Experiment 2**. Pre-training performance is defined as the mean performance during the first block of testing (before any training with feedback) in the first session. Post-training performance is defined as the mean performance of the last test block in the last session. Mean performance for sessions 1, 2, and 3 excludes the first block in the first session and last block in the third session. Error bars indicate the standard error of the mean across participants.

In Experiment 1, we compared learning between hard training (i.e., training to discriminate patterns at spiral angles of 35° vs. 55°) and easy training (i.e., training to discriminate patterns at spiral angles of 10° vs. 80°). Analysis of the training blocks (Figure 2A) showed that performance for the hard task improved significantly across training sessions [*F*(1.2,8.1) = 23.8, *p* < 0.01, Greenhouse–Geisser corrected], while performance for the easy task was at ceiling already for the first training session and did not improve significantly across sessions [*F*(1.1,7.5) = 3.6, *p* = 0.09, Greenhouse–Geisser corrected]. These results confirmed that discriminating patterns at spiral angles of 10° vs. 80° constitutes an easy task, while discriminating patterns at spiral angles of 35° vs. 55°constitutes a hard task that requires additional training. Further, analysis of the test blocks (Figure 2B) showed that for both groups (easy vs. hard training group), participants improved significantly in discriminating between similar Glass patterns presented at spiral angles of 35° vs. 55° (test blocks) after three sessions of training. In particular, a repeated-measures ANOVA showed a significant main effect of Session [Pre- vs. Post-training session, *F*(1,14) = 76.2, *p* < 0.01]. However, behavioral improvement was stronger following hard than easy training as shown by a significant interaction [*F*(1,14) = 9.8, *p* < 0.01] between-session (Pre- vs. Post-training) and Training task (Easy vs. Hard). No significant differences [*t*(14) < 1, *p* = 0.8] in performance were observed before training (i.e., first test block), suggesting that differences in post-training performance between easy and hard training could not be due to differences in baseline performance. These findings suggest that for the same amount of training, training on a hard discrimination results in better performance than training on an easy discrimination.

To test whether the lower improvement for easy compared to hard training was due to the limited amount of training (three sessions), we trained participants (*n* = 8) on the easy training task for six to eight sessions (Experiment 2). Participants improved across sessions [*F*(2.1,8.4) = 16.2, *p* < 0.01, Greenhouse–Geisser corrected] but performance saturated on average after the fifth session (Figure 2C). Comparing post-training performance for shorter (Experiment 1) and longer (Experiment 2) easy training protocols did not show any significant differences [*t*(14) = 1.11, *p* = 0.28]. Further, performance after longer easy training was significantly weaker than performance for hard training [*t*(14) = 2.8, *p* = 0.02], suggesting possible limits in behavioral improvement for easy training.

### Learning time course for hard vs. easy training

**Figure 3:**
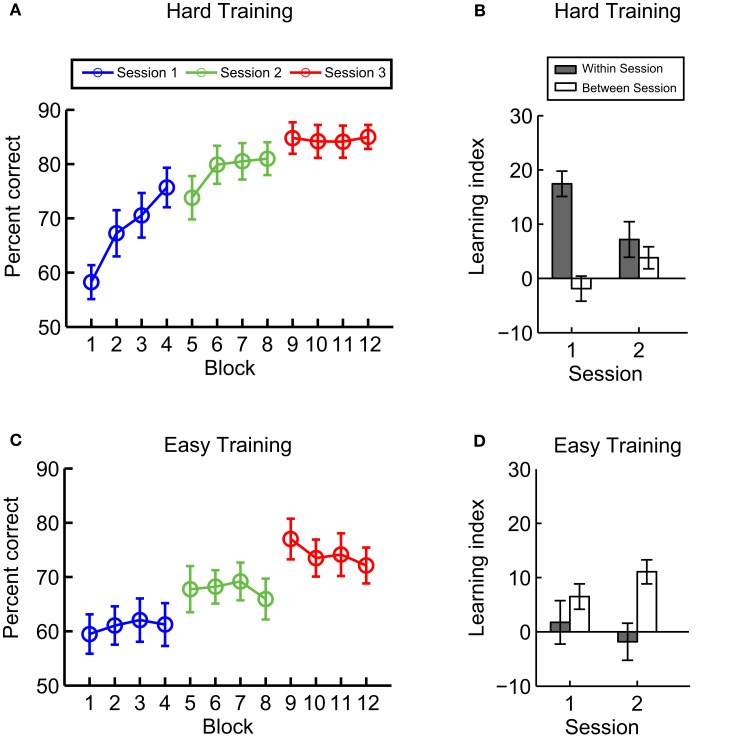
**Experiment 1: time course of learning**. Mean behavioral performance across participants per block for **(A)** hard and **(C)** easy training. We calculated within- and between-session learning indices **(B)** hard and **(D)** easy training. The within session index was calculated by subtracting the mean performance in the first test block from the last test block in a session. The between-session index was calculated as the mean performance difference between the last test block in the preceding session and first test block in the subsequent session. Error bars indicate the standard error of the mean across participants.

We investigated the time course of learning for hard vs. easy training by plotting the participants’ performance across test blocks in Experiment 1 (Figures 3A,C). We observed different time courses for the two training procedures. For hard training, discrimination performance increased within each of the first two sessions before reaching a plateau during the last session. In contrast, for the easy-training condition, there was no significant improvement within a session. However, performance increased between training sessions.

To quantify these observations, we defined within- and between-session learning indices. The within-session learning index was calculated by subtracting mean performance in the first test block from mean performance in the last test block in each session. The between-session learning index was defined as the mean performance difference between the last block in the preceding session and first block in the subsequent session. We calculated the within- and between-session learning indices for the first two sessions, as there was no subsequent session to calculate this index for the third session (Figures 3B,D). A repeated-measures ANOVA showed a significant interaction [*F*(1,14) = 10, *p* < 0.01] between learning index (Within vs. Between) and training task (Easy vs. Hard), consistent with stronger within-session learning for hard training [*F*(1,14) = 10.7, *p* < 0.01], while stronger between-session learning for easy training [*F*(1,14) = 7.7, *p* = 0.02].

### Experiment 3: Hard training with different stimuli than testing

**Figure 4:**
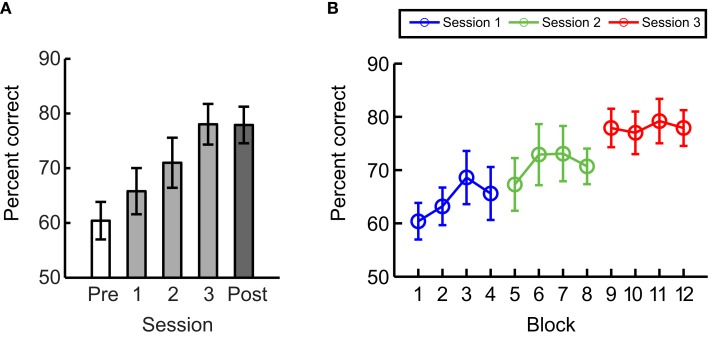
**Experiment 3**. Mean performance across participants per **(A)** session and **(B)** block when participants were trained with stimuli at spiral angles of 40° vs. 50° that differed from the test stimuli (35° vs. 55°). Error bars indicate the standard error of the mean across participants.

In Experiment 3, we trained and tested participants in a hard discrimination but with stimuli presented at different spiral angles. The aim of this experiment was to control for the possibility that performance differences between hard and easy training (Experiment 1) were due to the fact that participants in the hard training group were trained and tested with stimuli presented at the same spiral angle (35° vs. 55°) while participants in the easy training group where trained and tested with stimuli presented at different spiral angles. In particular, we tested participants with stimuli presented at spiral angles of 40° vs. 50° and tested with stimuli presented at spiral angles of 35° vs. 55°. We observed a similar pattern of results (Figure 4) as for hard training in Experiment 1 (Figure 3); that is, behavioral performance improved within each session (mainly sessions 1 and 2, but had saturated by session 3). In particular, a repeated-measures ANOVA showed no significant interaction [*F*(1,11) = 2.6, *p* = 0.14] between Learning Index (Within- vs. Between-session) and Experiment (Experiment 1 vs. 3). These results suggest that differences in the time course of learning for easy vs. hard training are due to differences in the difficulty of the training rather than simply the stimuli used for these two training protocols.

### Experiment 4: Learning without feedback

**Figure 5:**
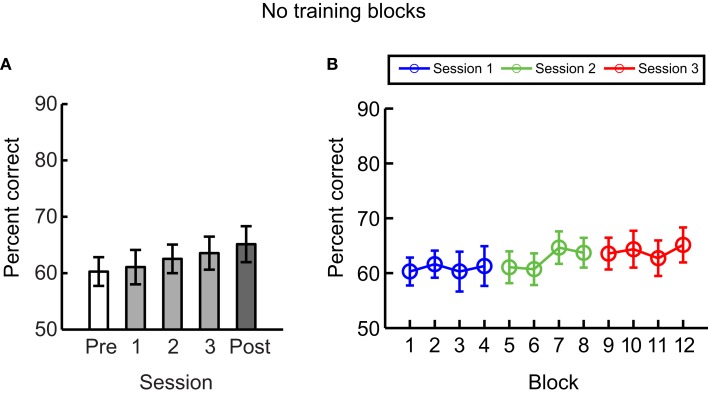
**Experiment 4**. Mean performance across participants per **(A)** session and per **(B)** block when only test but no training blocks were included in each session. Error bars indicate the standard error of the mean across participants.

To control for the possibility that improved performance could result from exposure to the test stimuli rather than training with feedback, we tested participants on the same number of test blocks (*n* = 4) as in Experiment 1 (i.e., participants were presented with stimuli at spiral angle of 35° vs. 55° without feedback) but did not expose them to any training blocks with feedback (Figure 5). Our results showed that observers’ performance did not improve significantly across sessions [*F*(4,24) = 1.8, *p* = 0.17] and post-training performance was significantly lower without (Experiment 4) than with (Experiment 1) training [*t*(13) = 5.2, *p* < 0.01]. These results suggest that training with feedback rather than mere exposure to the stimuli is required for improvement in the discrimination of similar global form patterns.

## Discussion

Our results demonstrate differences in visual shape learning and transfer performance depending on the difficulty of the training task. Training on a difficult task supports continuous and strong improvement in discriminating specific stimulus features that are similar between training and test. However, in the case of easy training, visual features differ between the stimuli used for training (i.e., highly discriminable) and test (i.e., highly similar) resulting in lower behavioral improvement that may require consolidation across sessions.

Our findings are consistent with previous work showing that introducing easy trials at the beginning of training facilitates subsequent training at more difficult conditions (Rubin et al., 1997; Liu, 1999; Liu and Weinshall, 2000). Training on an easy discrimination provides information about the global stimulus structure that may facilitate perceptual judgments by optimizing decision templates; that is, enhancing the behavioral relevance of features that are critical for the discrimination of global forms (Ahissar and Hochstein, 2004; Ahissar et al., 2009). Interestingly, easy training has been suggested to generalize more than hard training to untrained stimulus features (e.g., orientation) or visual field locations (Ahissar and Hochstein, 1996). These learning transfer effects suggest that easy training may alter higher-level processes related to the extraction and optimization of decision templates or rule-based learning (Zhang et al., 2010) for efficient perceptual judgments.

Computational models of perceptual learning offer interesting suggestions regarding the implementation of hard vs. easy training in the human brain. Perceptual learning is thought to occur by reweighting decision networks (Dosher and Lu, 1998, 1999; Adini and Sagi, 2001). It is possible that the optimal weights for the hard training and the hard test (i.e., discrimination of highly similar shapes) are more similar than the weights for the easy training and the hard transfer test. As a result, learning and transfer of different weight structures could have different time courses for easy vs. hard training. In particular, for hard training, local network weights could be adjusted through feedback based on specific parameters that match between training and test trials, as the stimulus features are similar between training and test stimuli. This weight adjustment may occur in real-time and affect local neural network outputs across trials, resulting in continuous performance improvement within single sessions (Petrov et al., 2005). In contrast, for easy training, stimulus features differ between training and test. As a result, real-time reweighting of local neural networks may not be possible due to the different optimal weight structures between the easy training task and the hard transfer test. However, as the majority of trials during easy training are judged correctly, the decision network can be optimized through Hebbian learning (i.e., stimulus-response associations). The optimization process is not limited to local neural networks based on trained stimulus parameters and may require a longer time period resulting in performance improvement across rather than within sessions.

It is also possible that sleep contributes to the consolidation of decision templates resulting in enhanced performance across sessions. Consolidation during sleep has been shown to enhance perceptual learning (Karni et al., 1994; Mednick et al., 2003). In particular, sleep has been suggested to be important for storing new information into long-term memory and strengthening of synaptic connections (Mednick et al., 2002), modulating learning specificity (Yotsumoto et al., 2009), and preventing disruption from training on other stimulus parameters (Seitz et al., 2005). Although in our study we did not manipulate or monitor the participants’ sleep patterns, it is possible that consolidation during sleep is more important for easy training that involves Hebbian learning of decision templates at larger scale networks than online reweighting of representations within session based on the trained stimulus features.

In sum, our results demonstrate differences in the time course of visual shape learning based on hard vs. easy training. Hard training may rely on online reweighting of shape representations based on trained features, while easy training entails transfer of learning from dissimilar to similar features and may require the optimization of decision templates or rule-based learning (Zhang et al., 2010). Understanding how the difficulty of the training task affects learning is of critical importance for the design of effective training programs that can be used in patient rehabilitation (e.g., Levi and Li, 2009). Our results show that easy training results in lower performance improvement compared to hard training. Even when participants were trained for at least double the number of trials, performance improvement was lower for easy than hard training. However, recent work suggests that mixing training trials from easy and hard conditions enhances learning without feedback, while feedback is required when participants are trained only with hard conditions (Liu et al., 2012). Thus, it is possible that interactions between hard and easy training may support more effective learning with potential applications in clinical practice.

## Conflict of Interest Statement

The authors declare that the research was conducted in the absence of any commercial or financial relationships that could be construed as a potential conflict of interest.
